# PRACTICE EFFECTS ON BRAIN EFFICIENCY OF WALKING IN AGING AND NEUROLOGICAL DISEASE

**DOI:** 10.1093/geroni/igad104.0550

**Published:** 2023-12-21

**Authors:** Roee Holtzer, Meltem Izzetoglu

**Affiliations:** Yeshiva University, New York City, New York, United States; Villanova University, Villanova, Pennsylvania, United States

## Abstract

Evidence provides support for using functional near-infrared spectroscopy (fNIRS) to quantify brain activations, notably in the pre-frontal cortex (PFC), under different walking conditions. Dual-tasks that involve walking impose additional demands on attention resources relative to the single tasks, and Studies using fNIRS revealed increased HbO2 in the PFC in Dual-Task-Walk (DTW) compared to Single-Task-Walk (STW) conditions in several populations. Burst measurement within session, which involves repeated administration of the same task, affords evaluation of short-term practice effects on changes in walking performance and the efficiency of its underlying neural activation. Here, we examined the effects of task (STW vs. DTW) and burst measurement (three counter-balanced repeated trials of each task condition) on walking performance and fNIRS-derived HbO2 in the PFC in older adults with multiple sclerosis (n=80, %female=65%, mean age=64ys) and controls (n=79, %female=65, mean age=69ys). Participants were enrolled in an ongoing cohort study designed to determine cognitive and brain predictors of locomotion in older adults with MS. Results from linear mixed effects models (LMEMs) revealed: 1) higher fNIRS-derived HbO2 in DTW compared to STW (p<.01). 2) fNIRS-derived HbO2 levels declined over repeated trials, notably in DTW (p<.01). 3) gait velocity was slower in DTW compared to STW (p<.01). 4) gait velocity improved over repeated trials (p<.01). The combination of decline in fNIRS-derived HbO2 levels and faster walking over repeated trials under DTW suggests more efficient usage of brain resources to support attention-demanding locomotion in aging and neurological disease.

